# Updated understanding of the protein – DNA recognition code used by C2H2 zinc finger proteins

**DOI:** 10.1016/j.sbi.2024.102836

**Published:** 2024-05-15

**Authors:** Xing Zhang, Robert M. Blumenthal, Xiaodong Cheng

**Affiliations:** 1 Department of Epigenetics and Molecular Carcinogenesis, University of Texas MD Anderson Cancer Center, Houston, TX 77030, USA; 2 Department of Medical Microbiology and Immunology, and Program in Bioinformatics, The University of Toledo College of Medicine and Life Sciences, Toledo, OH 43614, USA

**Keywords:** C2H2 zinc fingers, transcription factors, protein-DNA interactions, DNA sequence-specific recognition

## Abstract

C2H2 zinc-finger (ZF) proteins form the largest family of DNA-binding transcription factors coded by mammalian genomes. In a typical DNA-binding ZF module, there are twelve residues (numbered from −1 to −12) between the last zinc-coordinating cysteine and the first zinc-coordinating histidine. The established C2H2-ZF “recognition code” suggests that residues at positions −1, −4, and −7 recognize the 5′, central, and 3′ bases of a DNA base-pair triplet, respectively. Structural studies have highlighted that additional residues at positions −5 and −8 also play roles in specific DNA recognition. The presence of bulky and either charged or polar residues at these five positions determines specificity for given DNA bases: guanine is recognized by arginine, lysine, or histidine; adenine by asparagine or glutamine; thymine or 5-methylcytosine by glutamate; and unmodified cytosine by aspartate. This review discusses recent structural characterizations of C2H2-ZFs that add to our understanding of the principles underlying the C2H2-ZF recognition code.

## Introduction

DNA–protein interactions involving transcription factors (TFs) are essential for appropriate gene expression across the biological world [1-3], and they are gaining attention as druggable targets [4,5]. TFs act by binding at specific genomic locations. In eukaryotes, such binding is typically at gene promoters and/or enhancers, with some TFs binding only nucleosome-free regions and others capable of triggering alterations in chromatin structure [6,7]. To function properly, TFs recognize not only nucleosome content and modifications but also DNA supercoiling/bending [8,9] and epigenetic DNA modifications [10,11]. In mouse and human genomes, there are ~1500 annotated sequence-specific DNA-binding TFs [12,13]. Among these, three structural classes of DNA-binding proteins account for most TFs: Cys2-His2 (C2H2) zinc-finger (ZF) proteins (~700), homeodomain proteins (~250), and helix–loop–helix proteins (~100). Here, we focus on the C2H2 ZF-containing TFs and how they recognize specific DNA sequences.

**Figure 1:**
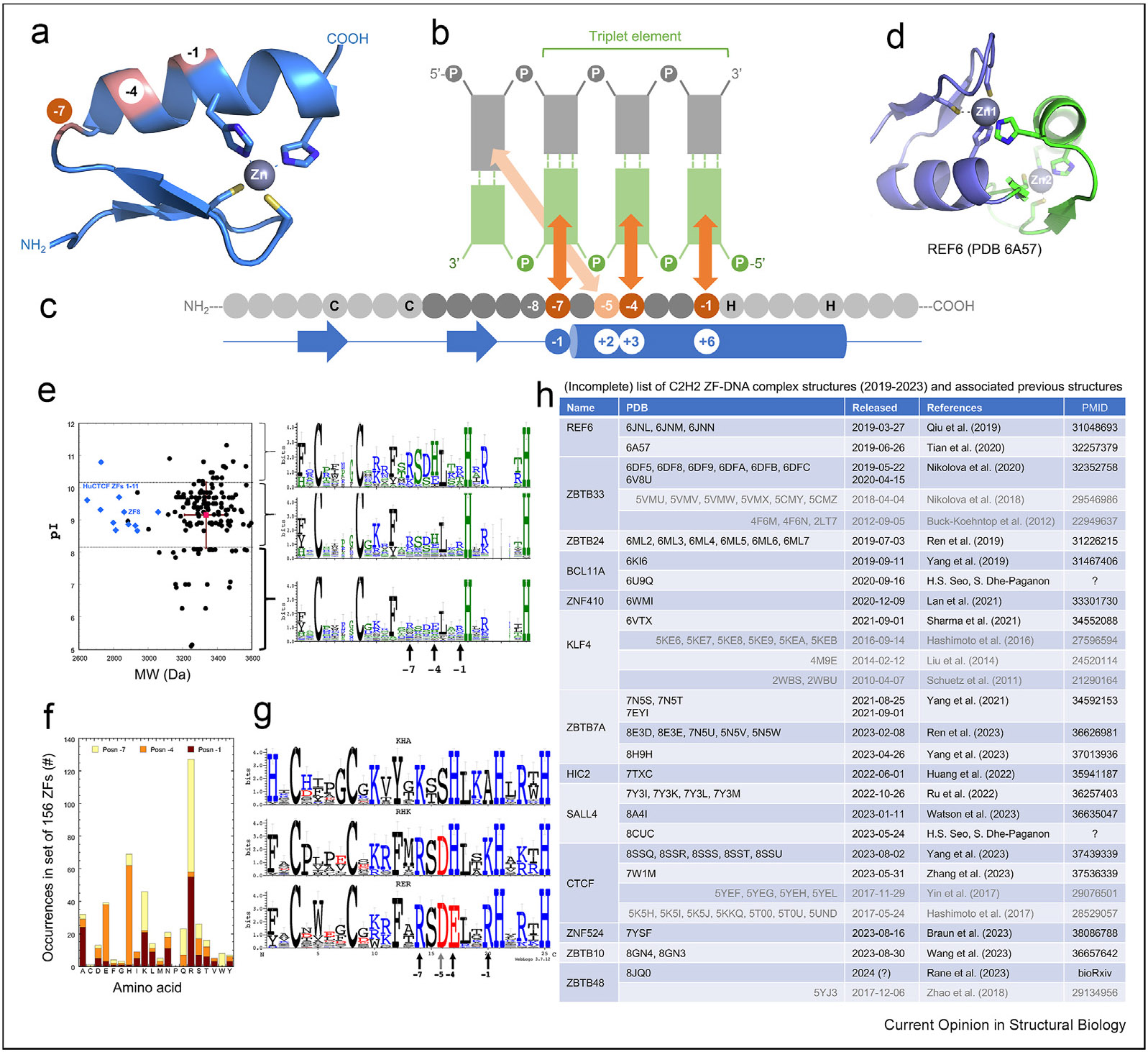
The canonical model of C2H2 ZF interaction with DNA triplet element. **(a)** Example of a C2H2 ZF module. The three residues at canonical positions of −1, −4, and −7 face the DNA. Two cysteine and two histidine residues in each finger (C2H2; in stick models) are responsible for one Zn^2+^-ion binding. [Residue numbering shown on the blue secondary structure diagram is described in panel c] **(b)** Schematic representation of a single ZF unit typically bound to three or four adjacent DNA base pairs via major groove contacts. The DNA sequence of the recognition strand (*bottom* in green) is oriented right to left from 5′ to 3′. The complementary strand (*top)* is colored in gray. **(c)** The protein sequence is from N-to-C termini (left-to-right) and amino acids at positions −1, −4, and −7 (highlighted) relative to the first Zn-associated histidine interact specifically with the DNA bases shown *above*. The protein secondary structures are shown below the sequence, with arrows for β strands, lines for loops, and cylinder for α helix. The traditional structure-based numbering at −1, +2, +3, and +6 (relative to the start of the α-helix) is provided for comparison. **(d)** A rare example of cross-finger zinc coordination in *Arabidopsis thaliana* REF6. **(e)** A set of 156 C2H2 ZFs was obtained from https://genexplain.com/tfclass/Class%202.3_alignment.html. The ZFs were from families 2.3.1 and 2.3.2. (*Left*) Distribution of pI *vs*. MW for each ZF, with the mean ± SD shown in red. For comparison, the 11 ZFs of human CTCF are shown as blue diamonds. (*Right*) The logos were generated using WebLogo3.0, after grouping the ZFs by pI as >1 SD above or below the mean, and those within 1 SD of the mean. The Zn ligands (2 Cys and 2 His) are invariant. The base recognition residues are indicated by vertical arrows. **(f)** Distribution of recognition amino acids at positions −7 (yellow), −4 (orange), and −1 (red) in this set are shown. **(g)** Logo analysis [86] of the ZF elements having the three most-frequent specificities (KHA, RHK, and RER at −7/−4/−1; vertical arrows). Together, these account for 66 of the 156 ZFs (42%). BHX (basic-His-any amino acid) should recognize the DNA sequence NGG; and RER (Arg-Glu-Arg) should recognize DNA sequence GMG, where M is Tor 5-methylC [40]. KHA triplet examples included ZF1 of Sp1 and ZF1 of KLF4. RHK triplet examples included ZF3 of Sp1 and ZF3 of KLF9. RER triplet examples included ZF2 of Sp1; ZF2 of KLF4; and both ZF1 and ZF3 of EGR1. **(h)** An (incomplete) list of recent C2H2 ZF-DNA complex structures discussed in this review. Abbreviations: CTCF = CCCTC-binding factor; MW = molecular weight (mass); ZF = zinc finger.

Conventional C2H2 ZFs are named for the Zn atom being coordinated by two Cys and two His residues, forming a Cys2–Zn–His2 tetrahedron that stiffens the fingers (Figure 1a) [14-17]. The term “fingers” has also been used for really interesting new gene (typically E3 ubiquitin ligases), plant homeodomain (typically histone lysine modification readers), Lin-ll, Isl-1, and Mec-3 (protein–protein interaction domains), and CCCH ZF RNA-binding proteins, among others [18-20]. These fingers differ in the number of zinc ions coordinated by different ligand combinations of Cys and His residues. Zn metalloproteins can also use Glu, Asp, Ser, and Thr to coordinate Zn [21,22], but to date, none of those four has been found to play this role in ZF proteins, probably reflecting the fact that, at physiological pH values, Cys and His have the highest affinities for Zn [23].

Here, we focus on the most frequently utilized DNA-binding C2H2 ZFs found among higher eukaryotic TFs [24,25]. There are ~700 mammalian C2H2 ZFs, and many of them also contain at least one protein–protein interaction domain of the classes KRAB, SCAN, BTB/ POZ, or SET, typically near their N-termini [26-30]. However, our focus here is on the DNA-binding C2H2 ZF arrays themselves, several of which have been examined experimentally to verify their DNA-binding motifs [31-34]. A longtime goal has been accurately predicting the sequence preferences of C2H2-ZFs directly from their amino acid (aa) sequences, to suggest testable biological functions. However, the canonical “recognition-code” model for C2H2-ZFs (Figure 1b) is based largely on specificity residues within well-defined three-finger arrays and remains incomplete and error-prone [35-39]. Possible reasons for this inconsistency include the following: a) the roles of aa outside of the 3–4 specificity residues within a single C2H2-ZF unit, b) the impact of neighboring C2H2-ZF fingers that function as major- or minor-groove spanners, and c) DNA conformation-modulated binding. In addition, human C2H2 ZF arrays contain 3 to ~35 fingers [40], leading to predictable binding sites as large as ~30–100 base pairs. These fingers do not necessarily all engage in DNA binding simultaneously, further complicating prediction of genomic binding sites. In this review, we discuss examples of C2H2-ZFs that violate the canonical C2H2-ZF “recognition code.” The resulting principles should help to improve predictions.

## ZF-position numbering used in this review

For C2H2-ZF proteins, the first structure reported for a three-finger protein complexed with DNA was Zif268/Egr1, more than three decades ago [41]. The DNA recognition process is sufficiently well understood to define a DNA recognition code [42,43], which, in turn, led to designed, sequence-specific ZF nucleases for genomic engineering [44-46]. This result implies a degree of modularity (independence between fingers and—within a finger—of the recognition aa), though such modularity is actually incomplete [39].

When bound to DNA, the helix of a typical ZF lies in the DNA major groove, whereas the antiparallel hairpin β strands and the C2–Zn–H2 unit lie on the outside, facing away from the DNA (Figure 1a). The N-terminal portion of each helix, and the first preceding residue prior to the helix, make major groove contacts with three adjacent DNA base pairs [42,43], which we term the “triplet element” (Figure 1b). Amino acids at positions −1, +3, and +6 (*bottom* of Figure 1c, in blue cylinder representing the α-helix) interact with the three bases of the recognition strand. In addition, the aa at position +2 interacts with the first base pair of the next triplet on the opposite strand (Figure 1b). This commonly used structure-based numbering scheme refers to the position immediately before the helix as −1, with positions +2, +3, and +6 within the helix. However, this numbering can lead to ambiguity (such as with the shorter helix in ZBTB7A [47]), so we use here the first zinc-coordinating His in each finger as reference position 0, with residues −1, −4, −5, and −7 corresponding to +6, +3, +2, and −1 of the structure-based numbering, respectively, (compare top and bottom in Figure 1c).

There are almost always 12 residues between the last zinc-coordinating Cys and the first zinc-coordinating His. This is much longer than the space between the two Cys ligands (2–4 residues) and between the two His ligands (3–4 residues). In addition, zinc coordination by three Cys and one His (C3H1) occurs in ZBTB7A ZF4 (CX_2_C-12 residues-HX_5_C) [47], and CCCTC-binding factor (CTCF) ZF11 (CX_2_C-12 residues-HX_3_C) [48-50]. We note that the CCHC-type ZF in the format of CX_2_C-4 residues-HX_4_C is common in RNA biology [51]. An unusual exception to the typical zinc coordination is *Arabidopsis thaliana* REF6, a DNA-sequence-specific histone lysine demethylase featuring four ZFs [52]. ZF1 lacks the final His ligand, which is provided by a His at position −6 of ZF2 (Figure 1d). This arrangement results in a compact structure of ZF1 and ZF2, but prevents ZF1–ZF2 of REF6 from binding DNA directly.

## C2H2-ZF DNA recognition code

The three aa of each finger at positions −1, −4 and −7 (relative to the first Zn-coordinating His) or +6, +3 and −1 (relative to the α-helix) are the principal determinants of DNA base recognition. These aa recognize (respectively) the 5′, central, and 3′ positions of each triplet, primarily on one DNA strand (the “recognition strand”). As represented in Figure 1c, the protein sequence runs left to right from N to C termini, whereas the DNA sequence of the recognition strand runs “antiparallel”, right to left from 5′ to 3′ . By analyzing a set of 156 ZFs from a family of C2H2 proteins, we note a range of masses and pI values (Figure 1e), but most strikingly, the −7/−4/−1 triplets of recognition aa are very strongly biased (Figure 1f). Specifically, a third of the set of 156 has the pattern BHX (where B = basic = Lys or Arg, H = His, and X = any residue), and another fifth of them has the pattern RER (Arg-Glu-Arg) (Figure 1g). While some of this may reflect repetitive ZF proteins such as KRAB-ZF, which apparently underwent duplication to recognize repetitive DNA sequences [29,31,53,54], there is substantial concentration (in this known family) of the triplets in the available sequence space. For example, Arg and Lys are preferred at −1 and −7, His and Glu at −4, and Ala at −1 (Figure 1f-g).

Bulky and charged/polar residues at base-interacting positions confer specificity for guanine (commonly by Arg, Lys, or His), adenine (by Asn or Gln), or cytosine (by Asp or Glu). These base-specific interactions are established for many protein–DNA interactions (including C2H2 ZFs [reviewed in Refs. [10,40,55-57]]. Thymine and 5-methylcytosine both contain a methyl group at pyrimidine ring carbon 5 and are recognized via either C–H…O-type interactions with Glu [40,56,58] or methyl-specific van der Waals contacts as illustrated by Zfp568 [59] and SALL4 [60,61] (discussed in the following).

When the −1, −4, and/or −7 base-interacting positions are occupied by small (Gly, Ala, Ser, Thr) or nonaromatic hydrophobic residues, the corresponding DNA sequence usually is a variation of the consensus sequence. The variable bases also form (water-mediated) hydrogen bonds (H-bonds) and van der Waals contacts with these aa. These contacts are “versatile,” in the sense they can not only recognize more than one base at a given position but also exclude one or more. This implies that the participating aa can suit the varied DNA substrates and intimately fit the ZF array to a variety of sequences. This adaptability to sequence differences applies to ZF arrays such as human PRDM9 at recombination hot spots [62], and CTCF at chromatin loops [48,49]. Here, we focus on recent examples of DNA-base-specific interactions engaging ZF residues (Figure 1h), at ‘nonclassic’ positions (particularly at positions −5 and −8) that are not part of the original canonical model, even in a large survey of the three-finger DNA-binding landscape [38] and in a recent deep-learning-based prediction [39].

## Arg – Asp switch at positions −8 and −7

**Figure 2:**
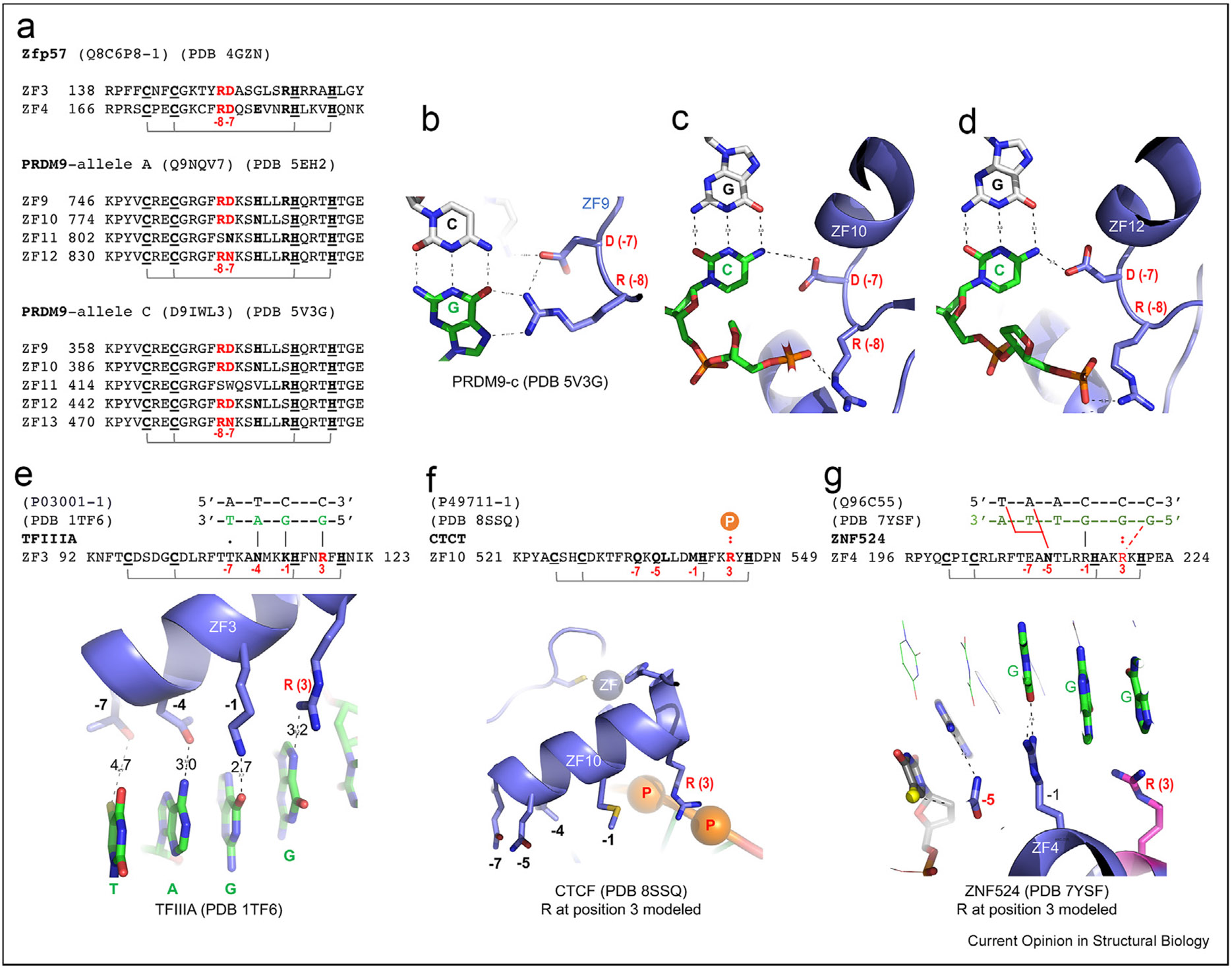
An Arg–Asp (RD) switch at positions −8 and −7. The DNA recognition strand bases are in green, with the complementary strand in gray. **(a)** Examples of ZFs containing RD at positions −8 and −7. **(b)** ZF9 of PRDM9 allele-C has R at −8 interacting with Gua. **(c–d)** ZF10 and ZF12 of PRDM9 allele-C have D at −7 interacting with Cyt. **(e)** The ZF3 of TFIIIA spans four base pairs with the Arg at position +3 between the two His ligands (Hxx**R**xH) interacting with guanine. **(f)** The (modeled) corresponding Arg at position 3 of ZF10 in CTCF could make DNA contacts with two neighboring phosphate groups. **(g)** The (modeled) corresponding Arg (magenta) at position 3 of ZF4 in ZNF524 could make DNA base contacts. Abbreviations: CTCF = CCCTC-binding factor; ZF = zinc finger.

Both Zfp57 (important for genomic imprinting) and PRDM9 (meiotic recombination) contain an N-terminal KRAB domain and, in the case of PRDM9, a SET domain [63,64]. An ArgeAsp (RD) dipeptide immediately precedes the ZF helix, at −8 and −7, in two neighboring Zfp57 fingers, as well as in two fingers of PRDM9 allele-A and three of allele-C (Figure 2a) [62,65,66]. Using PRDM9 allele-C as an example, with guanine in the recognition strand, the RD Arg forms bidentate H-bonds, whereas the Asp hydrogen-bonds with the Arg (Figure 2b). However, if the G:C base pair is inverted to C:G; these same RD residues adopt different conformations and partners. Specifically, the Asp now H-bonds with cytosine in the recognition strand, and, as a result, the adjacent Arg instead interacts with a backbone phosphate (Figure 2c-d). Thus, the RD dipeptide accommodates both C:G (G–Arg interaction) and G:C (C–Asp interaction). The same adaptability could apply to fingers that contain ArgeGlu (RE), Arg–Asn (RN), or Arg–Gln (RQ) at −8 and −7, allowing recognition of C:G (G–Arg) and G:5mC (5mC-Glu), or T:A (A–Asn) and T:A (A-Gln).

## Arg between two zinc-liganded His residues

TFIIIA of *Xenopus laevis* was one of the first C2H2 ZF proteins identified [14]. The crystal structure of the first six fingers of TFIIIA, bound with 31 bp of the 5S rRNA gene promoter, revealed that ZF1–ZF3 wrap around the major groove of DNA in the classic manner, whereas ZF4–ZF6 run along one side of the DNA, forming an open, extended structure [67] (we discuss ZF4–6 below). In ZF1–3, the space between the two Cys ligands is four residues (CX_4_C) and the space between the two His ligands is HX_3-4_H—either three residues (ZF1 and ZF2) or four (ZF3). ZF3 has a Lys, Asn, and Thr at base-interacting positions −1, −4, and −7 (Figure 2e). As expected, Lys at −1 interacts with a guanine and Asn at −4 with an adenine, but Thr (a small residue) at −7 is too far away to reach the corresponding base of the triplet element (Figure 2f). Interestingly, an Arg between the two His Zn ligands (Hx_2_**R**XH) interacts with the guanine 5′ to the triplet, which effectively shifts the recognized triplet by one bp, or expands the coverage of the ZF module to four bp (Figure 2f). This is a rare instance in which an Arg between two zinc-liganded His residues interacts base specifically. In most cases of HX_3_H we examined, positively charged residues between the two His ligands interact with DNA phosphates. However, as with the Hxx**R**XH motif in ZF3 of TFIIIA, ZF10 of CTCF [50] and ZF4 of ZNF524 [68] have Arg at the analogous position, yet in the two latter instances, the Arg is disordered. We constructed a model of the Arg side-chain, which led to the suggestion that in ZF10 of CTCF, this Arg likely makes contact with the DNA backbone (Figure 2g), whereas in ZF4 of ZNF524, it appears to engage in a base-specific contact (Figure 2h).

We searched a database of all human C2H2 ZF proteins, at smart.embl.de/smart/do_annotation.pl?DOMAIN=SM00355, for the subset containing HXX**R**XH motifs downstream of an appropriate CX_2-4_C pair, with a 12-residue spacer in between, and found 105 Hxx**R**XH motifs in 90 proteins. Interestingly, one of these proteins (ZNF142) has four of these Hxx**R**xH ZFs. Mutations of ZNF412 have been linked to neurodevelopment disorders [69-74]. In one study, among the 27 different *ZNF142* variants identified from 35 individuals, only four were missense [70] (S763C, C1233F, F1295L and R1500W of UniProt P52746). Among these, three residues are within a ZF unit: C1233F, involved in zinc coordination; F1295L, part of the hydrophobic core; and R1500W, involved in DNA backbone phosphate interactions.

## Small side-chain at position −5

**Figure 3:**
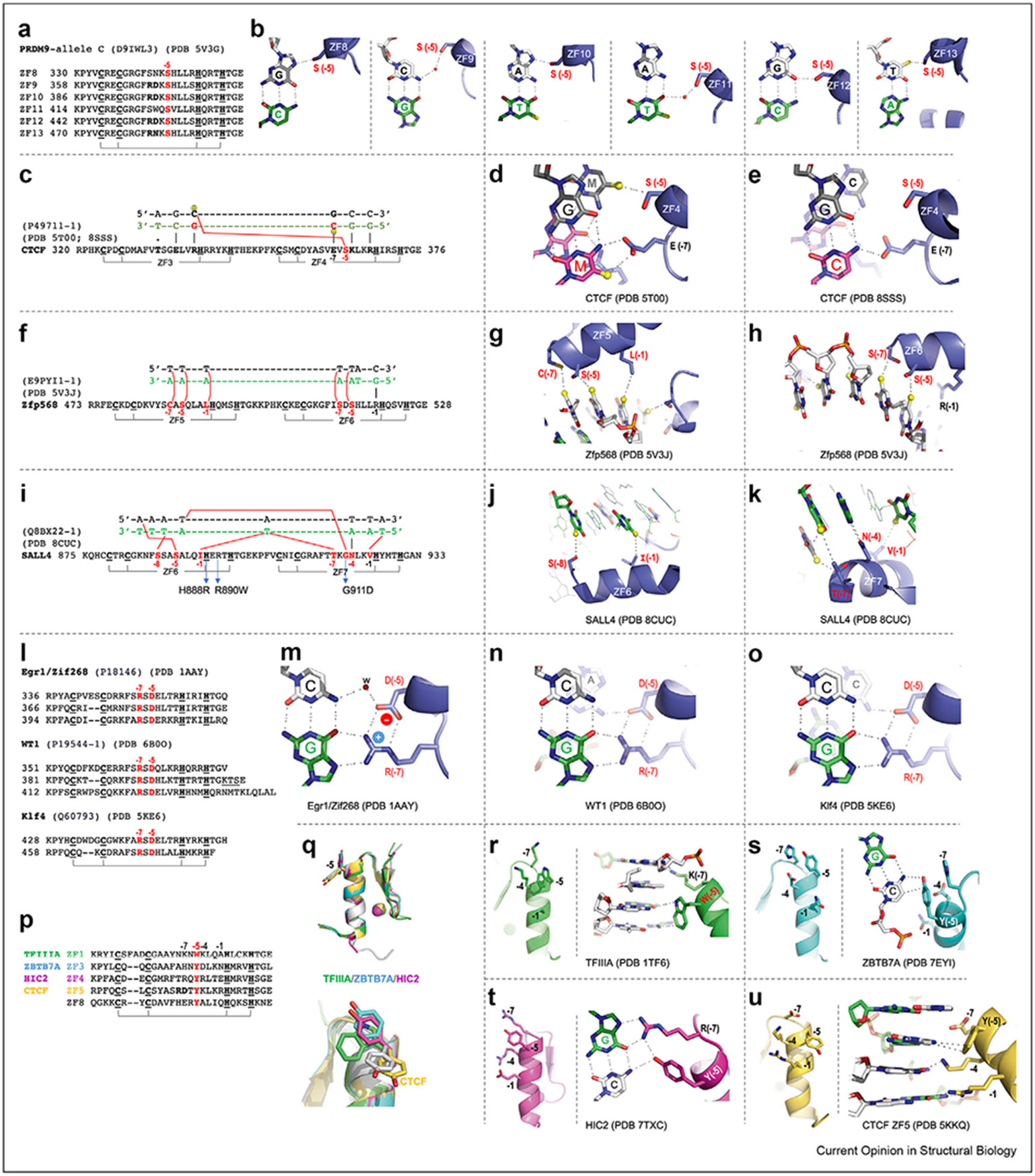
Varied residues at ZF position −5. **(a)** Sequence alignment of six C2H2 fingers of PRDM9 allele-C with invariant Ser at position −5. **(b)** The completely conserved Ser at position −5 in each ZF of PRDM9 interacts with DNA that differs from base pair to base pair. The DNA recognition strand is in green, and the complementary strand in gray. **(c)** A CpG dinucleotide is recognized jointly by ZF3 and ZF4 of CTCF. **(d)** CTCF uses Glu at position −7 of ZF4 to recognize 5-methylcytosine (5mC), and Ser at −5 to contact 5mC on the opposite strand. **(e)** The same CTCF ZF4 can also bind unmodified CpG. **(f)** Methyl-specific interaction with an A:T-rich sequence by Zfp568. **(g–h)** Zfp568 ZF5 and ZF6 interaction with five thymine bases (with methyl groups as yellow balls). **(i)** Methyl-specific interactions with A:T-rich sequence by SALL4. Patient missense mutations associated with Okihiro syndrome are indicated below the sequence. **(j–k)** SALL4 ZF6 and ZF7 interaction with five thymine bases (with methyl groups in yellow balls). **(l)** Examples of ZFs containing an Arg–Asp (R-D) pair at positions −7 and −5. **(m)** In Egr1/Zif268, the Asp at −5 of R-D pair interacts with the cross-strand cytosine via water-mediated interactions. **(n)** In WT1, the R-D Asp at −5 H-bonds with the cross-strand and cross-triplet adenine. **(o)** In Klf4, the R-D Asp H-bonds with the cross-strand and cross-triplet cytosine. **(p)** Examples of ZFs containing Trp or Tyr at position −5. **(q)** Two orthogonal views of superimposition of five fingers reveal two alternative conformations of Trp/Tyr at −5. **(r)** In TFIIIA, Trp at −5 points in the same direction as Lys at −4. **(s)** In ZBTB7A, Tyr at −5 points in the same direction as Asp at −4 and His at −7. **(t)** In HIC2, Tyr at −5 points in the same direction as Arg at −7. **(u)** In CTCF ZF5, Tyr at −5 points in the opposite direction to Lys at −4 and Arg at −1. Abbreviations: CTCF = CCCTC-binding factor; ZF = zinc finger.

In the canonical model of C2H2 ZFs [42,43], the small aa at the −5 position makes cross-triplet and cross-strand interactions with the first base pair of the following triplet (Figure 1b). Again, we use PRDM9 allele-C as an example (Figure 3a). Despite complete conservation of Ser at position −5 in each ZF in this case, the serines interact, sometimes via water-mediated contacts, with all four possible bases (Figure 3b). This adaptability stems in part from the ability of Ser (or other small aa) to act as an H-bond donor or acceptor, or both at the same time, or to provide van der Waals contacts. The cross-strand contact mediated by the small aa at −5 [corresponding to position 2 in structure-based numbering (Figure 1b, *bottom*)] is thus generally *not* a determinant of DNA-binding specificity.

Considering that both 5-methylcytosine (5mC) and thymine contain a methyl group at pyrimidine ring carbon 5, the small side-chain at position −5 could provide enhanced contact with 5mC at the CpG methylation site. Example includes ZF4 of CTCF (Figure 3c). ZF4 can bind five versions: CpG/CpG, 5mCpG/5mCpG, CpG/5mCpG, 5mCpG/CpG, and CpA/TpG. This rather catholic binding occurs via interactions with Glu at −7 position for the recognition strand (cytosine or 5mC), whereas Ser at −5 for the cross-strand interaction is promiscuous, accepting a thymine, 5mC or cytosine (Figure 3d-e).

In Zfp568, two fingers together (ZF5 and ZF6) recognize thymines of the opposite strand for a stretch of five A:T bases pairs (Figure 3f). Instead of the adenines of the recognition strand, the five thymines of the opposite strand are contacted by four small side-chains (Ser and Cys) and one hydrophobic residue (Leu) via van der Waals interactions with thymine methyl groups (Figure 3g-h). Such interactions, over a stretch of five bases of the nonrecognition strand, have not previously been described in classical ZF–DNA complexes.

A notable yet unexpected finding emerged from the structural analysis of SALL4 [60,61], mutations of which are linked to Okihiro syndrome. SALL4 is composed of seven ZFs, organized into three clusters. Particularly, the C-terminal cluster, comprising ZF6 and ZF7, exhibits a unique composition: only one conventional polar residue, Asn at −4 of ZF7, and the other positions typically involved in base interactions instead contain small (Gly, Ser, Thr) or hydrophobic residues (Ile, Val) (Figure 3i). Together, Ser at −8 and Ile at −1 in ZF6, and Thr at −7, Gly at −5, and Val at −1 in ZF7, make van der Waals contacts with five thymine methyl groups in an A:T-rich DNA sequence (Figure 3j-k). Three patient mutations linked to Okihiro syndrome are H888R of ZF6, involved in zinc coordination, R890W of ZF6, involved in DNA backbone phosphate interactions, and G911D of ZF7, involved in cross-strand binding of a thymine (Figure 3i).

## Arg – Asp pair at positions −7 and −5

The three-finger-binding domain of Egr1/Zif268 is one of the first, and best structurally-studied, C2H2 ZF proteins [41,75]. Each finger has an Asp at −5, which is almost always associated with an Arg at −7 (additional examples include Wilms tumor protein WT1 [76] and Krüppel-like factor Klf4 [77]) (Figure 3l). The Arg at −7 H-bonds with a guanine. Each of these Argeguanine contacts is stabilized by Asp at −5 – the Asp side-chain carboxylate is bent over, and both oxygens form an H-bond salt bridge with the Arg guanidinium (Figure 3m). This Asp?–Arg interaction may help position and stabilize the long Arg side-chain, strengthening the Arg–guanine contacts [78]. In addition, the Asp at −5 has one of its carboxylate oxygens within hydrogenbonding (or water-mediated) distance of a cross-strand and/or cross-triplet interaction with the first base pair of the following triplet (Figure 3m-o). However, as with Ser at −5, the cross-stand and cross-triplet hydrogen bond by the Asp at −5 does not in this case mediate base recognition (apparently H-bonding with the amino group at either Cyt-N4 or Ade-N6).

## Aromatic residue at position −5

There are several examples of an aromatic residue at −5, including Trp of TFIIIA ZF1 [67], and Tyr in several cases: ZBTB7A ZF3 [47,79], HIC2 ZF4 [80], and CTCF ZFs 5 and 8 [50] (Figure 3p). We superimposed the five Trp/Tyr-containing fingers, and the aromatic rings take two alternative conformations: either pointing in the same direction as base-interacting residues or pointing away from the DNA (Figure 3q). In TFIIIA, ZBTB7A, and HIC2, the aromatic residues point in the same general direction as the base-interacting positions (−1, −4, and −7). In TFIIIA, Trp at −5 spans two base pairs and the bulky side-chain displaces the associated ZF 4Å away from the DNA, as well as from the base-interacting Lys residues at −4 and −7 (Figure 3r). The long Lys side-chain at −7, displaced from the recognition strand, can still reach the DNA base on the opposite strand. Similarly, ZBTB7A has Tyr at −5, with Asn, Asp, and His at base-interacting positions (−1, −4, and −7). Each of these residues could in theory make base-specific interactions but do not, with Tyr forming a T-shaped stacking geometry with cytosine in the major groove, whereas the corresponding residues at base-interacting positions have side-chains too short to reach the corresponding DNA base (Figure 3s). As with ZBTB7A, the −5 Tyr in ZF4 of HIC2 points toward the DNA, together with the largest side-chain of Arg at −7 and contacts the same G:C base pair, forming a four-way interaction (Figure 3t).

CTCF has two fingers, ZF5 and ZF8, with Tyr at −5 (Figure 3p). Each of them takes a different conformation (Figure 3q) (we discuss CTCF ZF8 in the following). With Tyr of ZF5 pointing away from the side-chains at base-interacting positions (Figure 3u), Arg at position −1 and Lys at −4 (the two longest side-chains) can reach the guanine bases, whereas the shorter Asp at −7 allows a variable base without a direct base contact.

Among the four examples compared here (excepting CTCF ZF8), there are three consequences of having a bulky and aromatic residue at position −5: a) pointing away from the base-interacting residues and allowing them to contact the DNA (CTCF ZF5); b) pointing along with the base-interacting residues and preventing shorter side-chains at base-interacting positions from reaching the DNA (ZBTB7A ZF3); and c) pointing along but still allowing the longest side-chains (Arg and Lys) to interact with a DNA base (HIC2 and TFIIIA).

## Large and charged/polar residue at position −5: cross-strand base-specific interactions

**Figure 4:**
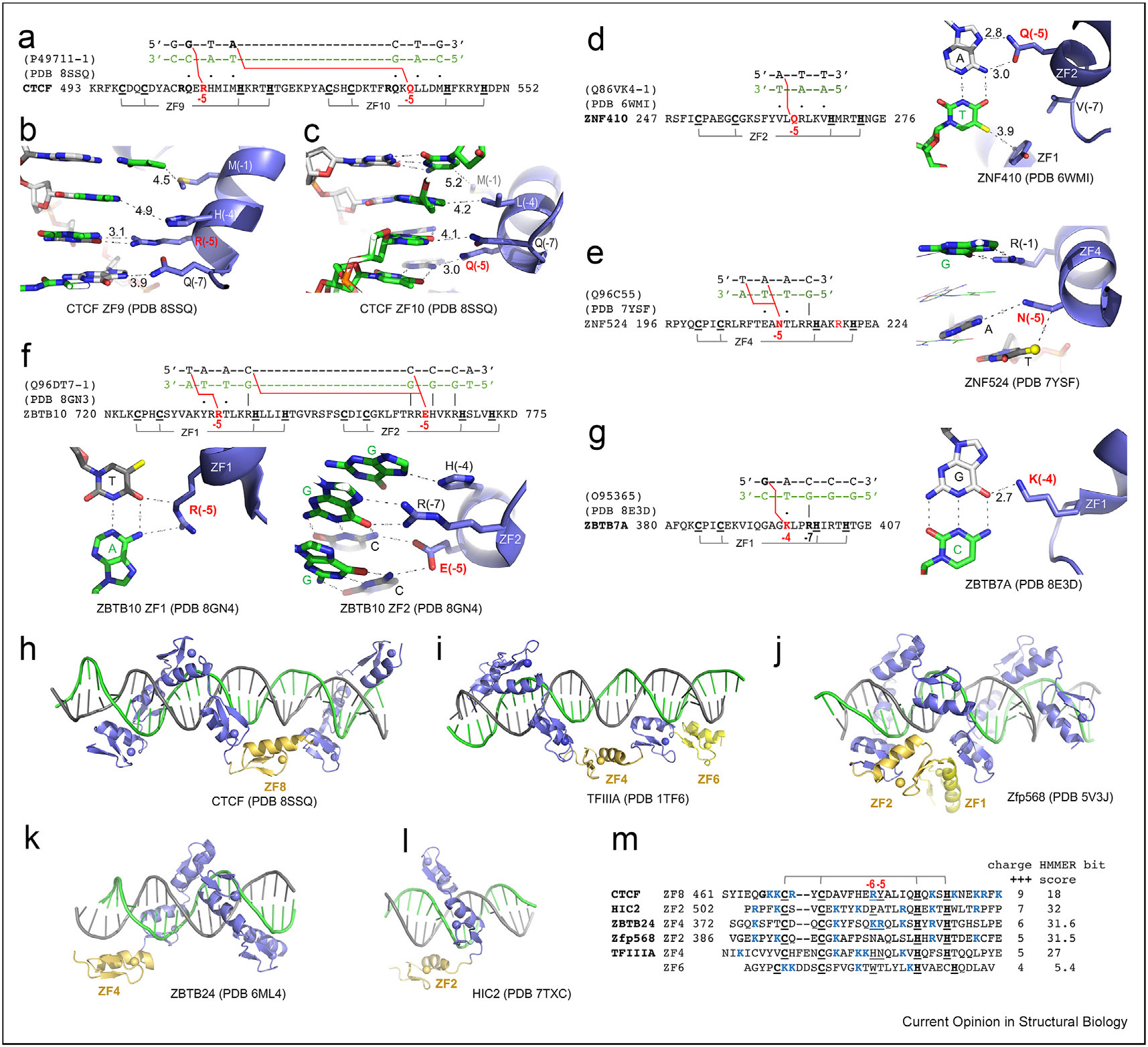
Large and charged/polar residue at position −5. **(a)** CTCF has an Arg at −5 of ZF9 and a Gln at −5 of ZF10. **(b)** A cross-strand guanine-specific interaction mediated by Arg at −5 of CTCF ZF9 increases the spacing distances between residues at −7, −4, and −1 and DNA. **(c)** A cross-strand adenine specific interaction mediated by Gln at −5 of CTCF ZF10 increases the spacing distances between residues at −7, −4 and −1 and DNA. **(d)** Gln at −5 of ZF2 in ZNF410 makes a cross-strand adenine-specific interaction. **(e)** Asn at −5 of ZF4 in ZNF524 makes cross-strand interactions with two adjacent TA bases. **(f)** Arg at −5 of ZF1 and Glu at −5 of ZF2 in ZBTB10 both make cross-strand interactions. **(g)** Lys at −4 of ZF1 in ZBTB7A makes a cross-strand guanine-specific interaction. **(h)** ZF8 of CTCF spans the minor groove. **(i)** ZF4 and ZF6 of TFIIIA are positioned across the minor groove. **(j)** ZF2 of Zfp568 spans the minor groove, while ZF1 is involved in inter-finger interactions. **(k)** ZF4 of ZBTB24 crosses the major groove without making base-specific contacts. **(l)** ZF2 of HIC2 traverses the minor groove. In all structures, the DNA recognition strand is in green and the complementary in gray. **(m)** Sequence alignment of six spacer ZFs including the pre- and post-linker regions. TFIIIA ZF6 has shorter linkers but expanded distances between the two Cys ligands and two His ligands of zinc ion. Abbreviations: CTCF = CCCTC-binding factor; ZF = zinc finger.

Continuing the theme of having a charged or polar residue at position −5: CTCF ZF9 and ZF10 have Arg or Gln, respectively (Figure 4a), whereas ZNF410 ZF2 has a Gln [81], ZNF524 ZF4 has an Asn [68], and ZBTB10 ZF1 and ZF2 have Arg or Glu, respectively [82]. Arg at −5 in CTCF ZF9 makes cross-strand interactions with guanine (Figure 4b). The large Arg side-chain pushes the α-helix of ZF9 away from the DNA interface, weakening the other interactions (increasing the distances) between DNA and His at −4 and Met at −1. Similarly, Gln at −5 of ZF10 makes a cross-strand interaction with adenine, weakening the interactions of Gln at −7, Leu at −4, and Met at −1 by increasing distances to their corresponding DNA bases (Figure 4c).

Other notable examples of cross-strand interactions involving residues at position −5 include: (i) a Gln at −5 of ZF2 in ZNF410, that forms a cross-strand interaction with an adenine base (Figure 4d), reminiscent of Gln in ZF10 of CTCF; (ii) an Asn in ZF4 of ZNF524 engages in cross-strand interactions with two adjacent bases in a TpA sequence (Figure 4e); and (iii) an Arg in ZF1 and a Glu in ZF2 of ZBTB10, both participate in cross-strand interactions (Figure 4f).

In sum, at least for the limited number of examples discussed here, larger side-chains (Arg, Gln/Asn, and Glu) at −5 make cross-strand base-specific interactions with guanine, adenine, and cytosine and provide base specificity for the corresponding C:G, T:A, and G:C base pairs, respectively. This situation is different from the “versatile” contacts made by smaller side-chains (*e.g*. Ser) at position −5, or an R-D pair at −7 and −5, as discussed earlier. Our analysis revealed that highly specific Arg–Gua and Gln/Asn–Ade interactions, recognizing G:C or A:T base pairs from canonical positions −1, −4, and −7, also apply to position −5 but in a cross-strand fashion. Base-specific contacts by larger and charged/polar residues at position −5 might compensate for cases, where small or hydrophobic residues at the cognate positions −1, −4, and −7 cannot provide specificity (*e.g*. Met at −1 and Leu at −4 of ZF10 in CTCF, and Val at both −1 and −7 of ZF2 in ZNF410).

Additionally, a cross-strand interaction between Lys and guanine has been observed in various instances, such as Lys413 of ZF1 in KLF4 [77], Lys328 of ZF4 in ZNF410 [81], and Lys396 of ZF1 in ZBTB7A [47,79] (Figure 4g). These Lys residues, unlike Arg or Gln at position −5, are situated at the conventional base-interacting locations of −7 (as in KLF4), −4 (as in ZBTB7A), or −1 (as in ZNF410). For instance, the ability of ZBTB7A Lys396 to switch from interacting with G_1_ in a G_1_ACCC sequence to G_2_ on the opposite strand in a GC_2_CCC sequence enables ZBTB7A to bind to sequences with variations at the second position in the G(a/c)CCC pattern [79].

## ZF as a spacer

In CTCF, instead of continuing in the DNA major groove, ZF8 spans the minor groove and acts as a spacer to properly position the C-terminal fingers ZF9–ZF11 in the major groove (Figure 4h) [48-50]. As ZF8 of CTCF, similar spacers have been seen in TFIIIA [67], in which ZF4 and ZF6 are positioned and across the minor groove to span the entire length of the duplex (Figure 4i); whereas in Zfp568 [59], ZF2 spans the DNA minor groove at an A:T-rich stretch (where the minor groove is narrower [59]) (Figure 4j); in ZBTB24 [83], in which ZF4 spans the DNA major groove, and in HIC2, where ZF2 spans the DNA minor groove (Figure 4k-l). There is no clear sequence similarity among the six examples of spacer ZFs (Figure 4m). CTCF ZF8 and its associated prelinker and postlinker regions harbor the largest number of positively charged residues (nine), which could interact with negatively charged DNA-backbone phosphates, followed by HIC2 (seven) and ZBTB24 (six). In addition, large and charged side-chains occupy the corresponding positions −6 and −5 in CTCF ZF8 (RY), ZBTB24 ZF4 (KR), and TFIIIA ZF4 (HN) (Figure 4m). HIC2 ZF2 and TFIIIA ZF6 contain a Pro (in the beginning of the helix) or Trp at position −6, and both are infrequent among the ZFs we examined (see Figure 1). We used the HMMER algorithm (http://zf.princeton.edu/index.php) to calculate the bit score for each finger, and all of them (except TFIIIA ZF6) have fairly high confidence scores, the lowest being 18 and the highest being 32. ZF6 of TFIIIA has shorter linkers (4–5 instead of 7–8 residues) but expanded distances between the two Cys ligands (5 instead of 2–4 residues) and two His ligands (4 instead of 3 residues) (Figure 4m). Nevertheless, it remains to be determined exactly what features would allow prediction of which ZFs could function as spacers.

## Summary and perspective

Seven large and charged or polar residues (Arg, Lys, His, Gln, Asn, Glu, and Asp) are commonly involved in DNA base-specific interactions within the major groove [84]. These interactions are particularly relevant when these residues are positioned at five specific locations in C2H2-ZF proteins: −1, −4, −5, −7, or −8 (Figure 1c). Their presence at these sites confers sequence specificity for both strands of the DNA. Additionally, small and nonaromatic hydrophobic residues at these positions often provide “versatile” contacts, which can enhance binding affinity, and in some instances, these residues make van der Waals interactions with the methyl group of thymine within A:T-rich sequences. Further research is required to fully understand the determinants that guide C2H2-ZFs to function as major- or minor-groove spanners or spacers, to induce DNA conformation changes upon binding, and to fully characterize the role of residues outside of these five specificity-conferring residues within a single C2H2-ZF unit (*e.g.* the Arg within Hxx**R**XH of ZF3 in TFIIIA).

In a significant breakthrough at the end of 2023, the food and drug administration (FDA) granted approval to the first clustered regularly interspaced short palindromic repeats (CRISPR) gene-editing treatment for sickle cell disease (FDA announcement: FDA Approves First Gene Therapies to Treat Patients with Sickle Cell Disease). This innovative treatment targets the *BCL11A* gene, which codes for a C2H2 ZF protein. BCL11A binds directly to the promoter of the fetal hemoglobin gene [85], playing a key role in the suppression of its production soon after birth. The CRISPR-mediated disruption of *BCL11A* activity facilitates the reactivation of fetal hemoglobin production, offering a promising new strategy for the management of sickle cell disease. This development underscores the vast, yet largely untapped potential of the approximately 700 mammalian C2H2 ZF proteins. Their diverse functions and roles in various biological processes offer numerous possibilities for future research and therapeutic interventions.

## Data Availability

No new data were generated for the research described in the article.
